# Cortical drive of low-frequency oscillations in the human nucleus accumbens during action selection

**DOI:** 10.1152/jn.00988.2014

**Published:** 2015-04-15

**Authors:** Max-Philipp Stenner, Vladimir Litvak, Robb B. Rutledge, Tino Zaehle, Friedhelm C. Schmitt, Jürgen Voges, Hans-Jochen Heinze, Raymond J. Dolan

**Affiliations:** ^1^Wellcome Trust Centre for Neuroimaging, University College London, London, United Kingdom;; ^2^Department of Neurology, Otto-von-Guericke-University Magdeburg, Magdeburg, Germany;; ^3^Department of Stereotactic Neurosurgery, Otto-von-Guericke-University Magdeburg, Magdeburg, Germany;; ^4^Department of Behavioral Neurology, Leibniz Institute for Neurobiology, Magdeburg, Germany; and; ^5^Max Planck University College London Centre for Computational Psychiatry and Ageing Research, London, United Kingdom

**Keywords:** nucleus accumbens, action selection, local field potentials, synchronization, deep brain stimulation

## Abstract

The nucleus accumbens is thought to contribute to action selection by integrating behaviorally relevant information from multiple regions, including prefrontal cortex. Studies in rodents suggest that information flow to the nucleus accumbens may be regulated via task-dependent oscillatory coupling between regions. During instrumental behavior, local field potentials (LFP) in the rat nucleus accumbens and prefrontal cortex are coupled at delta frequencies (Gruber AJ, Hussain RJ, O'Donnell P. *PLoS One* 4: e5062, 2009), possibly mediating suppression of afferent input from other areas and thereby supporting cortical control (Calhoon GG, O'Donnell P. *Neuron* 78: 181–190, 2013). In this report, we demonstrate low-frequency cortico-accumbens coupling in humans, both at rest and during a decision-making task. We recorded LFP from the nucleus accumbens in six epilepsy patients who underwent implantation of deep brain stimulation electrodes. All patients showed significant coherence and phase-synchronization between LFP and surface EEG at delta and low theta frequencies. Although the direction of this coupling as indexed by Granger causality varied between subjects in the resting-state data, all patients showed a cortical drive of the nucleus accumbens during action selection in a decision-making task. In three patients this was accompanied by a significant coherence increase over baseline. Our results suggest that low-frequency cortico-accumbens coupling represents a highly conserved regulatory mechanism for action selection.

the nucleus accumbens contributes to the selection of goal-directed actions by integrating behaviorally relevant information from the hippocampus, amygdala, and prefrontal cortex, among other areas (Goto and Grace 2008; Grace 2000). These inputs are likely integrated as a function of current task requirements (Calhoon and O'Donnell 2013; Gruber et al. 2009a; Stuber 2013). Previous work suggests that this task-dependent integration may be regulated by inter-area coupling of oscillatory synaptic potentials. In rodents, theta oscillations (around 7 Hz) in the ventromedial striatum (which includes the nucleus accumbens) are coherent with hippocampal theta oscillations during active exploration (Gruber et al. 2009a) and in maze tasks (Berke et al. 2004; van der Meer and Redish 2011). Entrainment of ventral striatal single units to the hippocampal theta rhythm has been associated with combined contextual (spatial) and motivational information (Tabuchi et al. 2000; van der Meer and Redish 2011). Together, these results have led to the proposal that theta coupling between the hippocampus and the ventral striatum/nucleus accumbens may regulate how contextual information influences action selection (Grace 2000; Gruber et al. 2009a; Pennartz et al. 2009).

Instrumental behavior, on the other hand, has been associated with oscillatory coupling of the nucleus accumbens with prefrontal cortex at delta frequencies (1–4 Hz). Gruber et al. (2009a) found strong delta coherence between the nucleus accumbens core and prefrontal cortex, accompanied by enhanced single-unit cross-correlation, when rats engaged in instrumental behavior compared with active exploration. Because theta coherence between the nucleus accumbens core and the hippocampus was simultaneously reduced, the authors suggested that cortico-accumbens coupling may override a hippocampal influence on neuronal excitability in the nucleus accumbens (Calhoon and O'Donnell 2013), thereby promoting behavioral flexibility. Translation of these findings to humans has remained speculative, largely because electrophysiological data from the human nucleus accumbens are rare. Such data have recently become more tractable via recordings from deep brain stimulation (DBS) electrodes, implanted in the nucleus accumbens for treatment of neurological or neuropsychiatric disorders.

In the present study, we examined functional and directed connectivity between cortex and the nucleus accumbens in six patients treated with DBS for drug-resistant epilepsy. We studied coherence, phase-synchronization, and Granger causality both at rest and during a value-based decision-making task. Given reports of cortico-accumbens connectivity at delta frequencies in rodents (Gruber et al. 2009a), we expected to find low-frequency coupling between cortical EEG and local field potentials (LFP) in the nucleus accumbens. Furthermore, we predicted that the strength of this coupling would increase during the action selection stage of our decision-making task, accompanied by evidence for a cortical drive of low-frequency oscillations in the nucleus accumbens, as demonstrated in rodents (Calhoon and O'Donnell 2013; Gruber et al. 2009a). Scalp recordings may reflect hippocampal signals in addition to neocortical signals (e.g., Kaplan et al. 2012). In this study, we could rule out a substantial hippocampal contribution to the observed LFP/EEG coupling in two of the patients who had undergone unilateral resection of the hippocampus before DBS surgery.

## METHODS

### Patients

Six patients with pharmacoresistant partial epilepsy participated in the study (age: 39.5 ± 8.6 yr, mean ± SD; 3 women; all right handed; see Table 1 for details). All patients participated in in-house protocols aimed to objectify safety and potential anti-ictal efficacy of deep brain stimulation of the nucleus accumbens. Clinical outcomes have been reported elsewhere (Kowski et al. 2015; Schmitt et al. 2014). Both this clinical trial and the experiments reported presently were approved by the institutional review board of the University of Magdeburg (registration number 03/08), and all patients gave written informed consent.

### Electrode Implantation

All patients underwent stereotactically guided surgery with implantation of quadripolar electrodes in the nucleus accumbens and anterior thalamus of each hemisphere (model 3387; Medtronic). Planning of electrode placement and surgical procedures were performed as described in detail in previous work (Voges et al. 2002; Zaehle et al. 2013). For the nucleus accumbens, standardized coordinates were 2 mm rostral to the anterior border of the anterior commissure at the level of the midsagittal plane, 3–4 mm ventral and 6–8 mm lateral to the midline. For each patient, these standardized coordinates were adjusted according to presurgical MRI, taking into account the anatomy of the vertical limb of Broca's diagonal band as an important landmark. Specifically, electrodes were placed 2–2.5 mm lateral to the vertical limb of Broca's band, in the caudomedial part of the nucleus accumbens. This area is thought to correspond to the remnant of the shell area in primates (Sturm et al. 2003), as defined by histochemical criteria. The position of each electrode was confirmed by intraoperative stereotactic X-ray images and postoperative computed tomography (CT). At least two contacts of each quadripolar electrode were placed inside the nucleus accumbens, covering those parts equivalent to the nucleus accumbens shell and core regions in rodents.

After surgery, electrode leads were externalized for 6 days, allowing test stimulation with different parameters as well as recordings from depth electrodes in various psychological tasks and at rest. Subsequently, electrode cables were connected to an impulse generator located beneath the left pectoral muscle.

### Procedure and Task

**Fig. 1.**
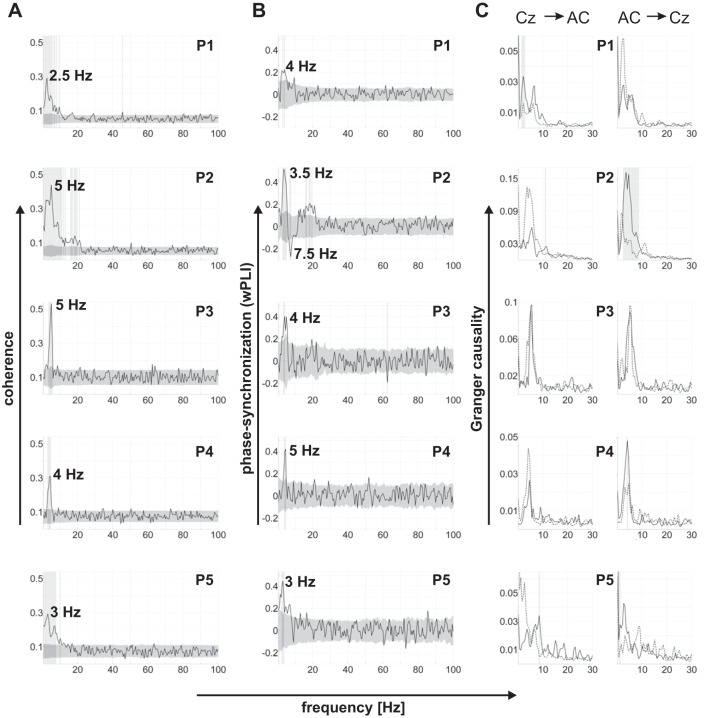
Coherence, phase synchronization, and Granger causality between cortex (EEG surface electrode at Cz) and the nucleus accumbens in resting-state data. Each row shows data for 1 of the 5 patients (P1 to P5). All *x*-axes represent frequency in Hz. The light gray vertical shading indicates frequency bins at which the original spectrum (black line) is significantly different from the trial-shuffled spectra (*A* and *B*) or the time-reversed spectrum (*C*; dotted line). The dark gray shading in *A* and *B* corresponds to 2 standard deviations around the mean of the trial-shuffled spectra. Numbers next to peaks correspond to peak frequencies. Data are coherence (*A*) and the weighted phase-lag index (wPLI), a measure of (non-zero) phase synchronization (*B*), for 5 patients. In *C*, the *left* column shows Granger causality spectra representing a cortical drive of the nucleus accumbens, whereas the *right* column shows coupling in the opposite direction (nucleus accumbens leading cortex). All patients show significant peaks in the coherence and wPLI spectra at low (delta and theta) frequencies (*A* and *B*). The direction of cortico-accumbens coupling at rest varies across patients (*C*). AC, nucleus accumbens.

**Fig. 2.**
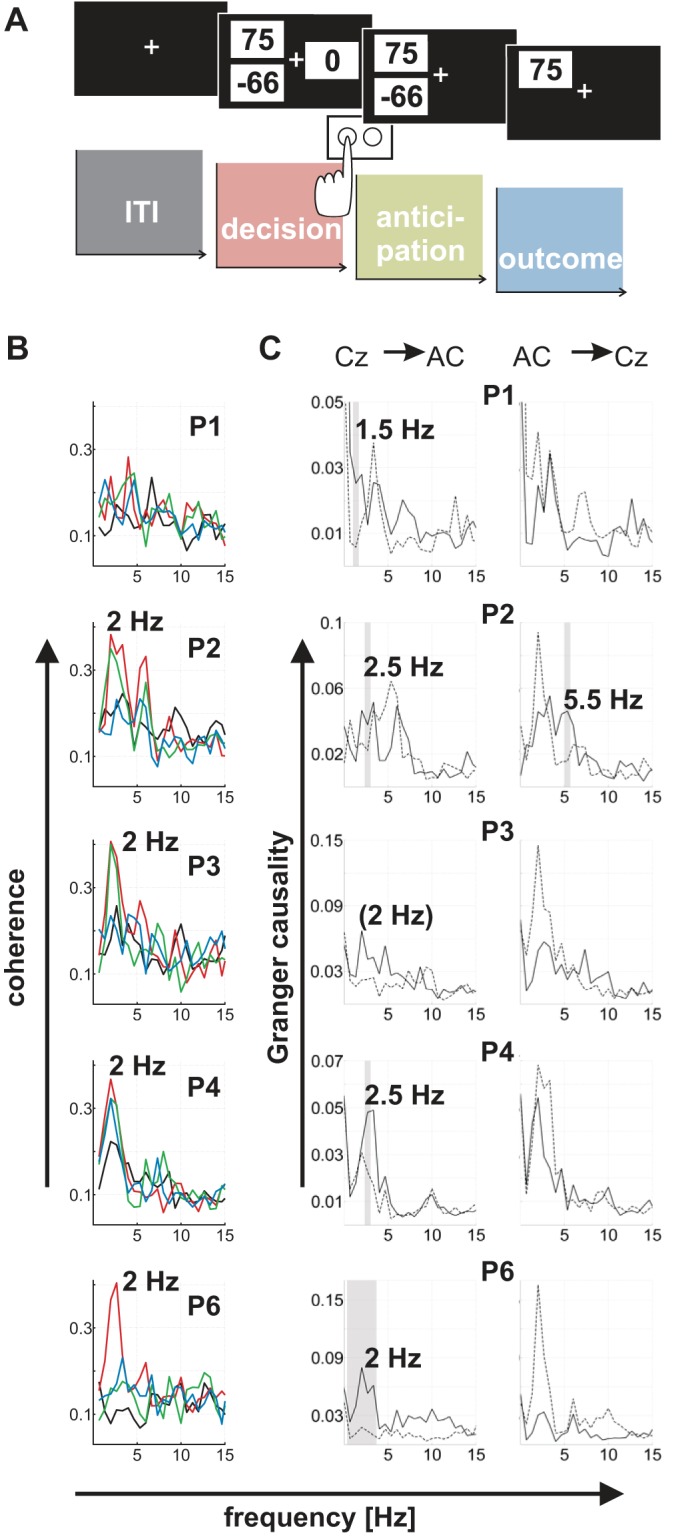
Coherence and Granger causality between cortex and the nucleus accumbens during the decision-making task. *A*: schematic of 1 trial of the task with distinct task stages (all intervals are equal to 1.5 s in duration). ITI, intertrial interval. *B*: coherence as a function of frequency during all 4 stages of the task, color-coded as in *A* (black, baseline/ITI; red, decision stage; green, anticipation stage; blue, outcome stage). Coherence is significantly enhanced during the decision stage compared with baseline in 3 patients (P2, P3, and P6). Numbers next to peaks correspond to peak frequencies. Each row shows data for 1 of the 5 patients (P1 to P6). *C*: Granger causality spectra for the decision stage of the task. As in Fig. 1*C*, the *left* column shows a Granger causal influence of cortex on the nucleus accumbens, whereas the *right* column shows coupling in the opposite direction (nucleus accumbens leading cortex). The dotted line shows Granger causality after the time axis is reversed at all channels. The gray vertical shading indicates frequency bins at which the original spectrum (black line) is significantly greater than the spectrum computed after the time axis is reversed. Four of the 5 patients show a significant cortical drive of delta oscillations in the nucleus accumbens (trend in P3).

Data were collected on two separate days between the third and fifth days postsurgery, with one data set collected at rest (Fig. 1) and one collected during a decision-making task (Fig. 2). Four patients contributed both resting-state data and task-related data. For one patient (P5), only resting-state data were collected, and for another patient (P6), only task-related data were collected. Consequently, each data set comprised five patients. For the resting-state recordings, patients sat comfortably in an upright position and were asked to remain as still as possible, with their eyes open. Three to ten minutes of resting-state data were recorded.

The task data set was recorded during a standard economic decision-making task, presented on a laptop computer. The nucleus accumbens is active in a variety of tasks that involve action selection (Nicola 2007). We used a task for which strong responses are reliably recorded from the human nucleus accumbens during action selection in previous functional MRI studies (Rutledge et al. 2014; Tom et al. 2007). Activity of single neurons in the nucleus accumbens can be used to predict subsequent actions in a financial decision-making task (Patel et al. 2012), even after controlling for the probability of reward. Because the objective value of the gamble in our task changes from trial to trial, making appropriate actions requires subjects to determine the subjective value of each available gamble. This makes such an economic decision task ideal for studying goal-directed actions. Furthermore, this task allowed us to additionally test for value and reward prediction error signals that might relate to dopamine, which have been identified in this area using other techniques (Hart et al. 2014; Patel et al. 2012). These investigations are the subject of a separate study.

The task required patients to decide whether to accept or reject a monetary gamble offer. If accepted, a gamble could result in a monetary gain or loss with equal probabilities. Each patient made 200 choices between this risky gamble option and a safe option, which was worth 0 euros. There were five different win amounts for the risky option (25, 40, 55, 75, or 100 euro cents). Loss amounts for the risky option (5–200 euro cents) were determined by multiplying gain amounts by 20 different multipliers, ranging from 0.5 to 5 to accommodate a wide range of gain-loss sensitivity. The risky gamble option and the safe option were represented by three numbers on the screen, each presented in a black font in the center of a white rectangle (see Fig. 2*A*; screen background color was black). Two of the three numbers were presented on one side of the screen (left or right) and corresponded to the possible gain and loss of the current gamble offer. The potential loss amount, indicated by a negative sign, and the possible win amount were presented slightly below and above the horizontal meridian of the screen, respectively, at equal eccentricities from the center of the screen. The third number, representing the safe option, was always zero and was presented on the opposite side of the screen, on the horizontal meridian. The side of the screen (left or right) on which each of the two options (safe and gamble) was presented was counterbalanced across trials.

Each trial started with the presentation of the two options on the screen. Patients chose the option presented on the left or on the right by pressing one of two keys on a keyboard with their left or right hand, respectively (left “Ctrl” and right “Enter” on the number block, respectively). There was no time limit for this decision. If patients chose to gamble, the outcome was randomly determined by the computer and displayed after a 2-s delay period for 1.5 s. The intertrial interval (ITI) was jittered between 1.5 and 2 s. The outcome of each trial counted for real money. Subjects were endowed with 15 euros at the start of the experiment and, in addition to this endowment, earned an average of 15.66 euros (range: 3.73–27.38 euros), which was paid out at the end of the experiment. Before the main experiment, patients practiced the task and became familiar with the range of possible wins and losses for 50 trials, which did not count for real money.

### Recording and Analyses of LFP

#### LFP and surface EEG recording.

LFP were recorded continuously from four contacts on each of the four DBS electrodes (one in the nucleus accumbens and one in the anterior thalamic nucleus of each hemisphere). Electrode contacts (platinum-iridium) were 1.5 mm wide and spaced 1.5 mm apart (edge-to-edge distance). Simultaneous with LFP recordings, data from two to five surface EEG electrodes (gold-plated silver electrodes) were also recorded (no online filters). Surface electrode number and placement varied between patients due to the presence of postsurgical head bandages. The only surface EEG channel that was available across all patients and both data sets was Cz (positioned according to the 10–20 system).

Data were digitized at a sampling rate of 512 Hz using a Walter Graphtek system. Following previous work (Litvak et al. 2012), we analyzed LFP data from DBS electrodes in a bipolar montage, in which each contact is referenced to one neighboring contact. This results in three channels per DBS electrode (i.e., each of the 3 most ventral contacts was referenced against its dorsal neighbor). This bipolar montage maximizes spatial specificity for the nucleus accumbens by minimizing volume conduction effects from distant sources. Surface EEG channels were referenced against the left ear lobe.

#### Preprocessing.

LFP and surface EEG data were analyzed using FieldTrip (Oostenveld et al. 2011) and MATLAB (version R2012a; The MathWorks). Line noise was removed using a narrow-band bandstop filter (4th-order, 2-pass Butterworth filter). Visual inspection showed a slow drift at the very beginning of the resting-state data, likely due to a long time constant of the amplifier. This drift was removed using a high-pass filter (6th-order, 2-pass Butterworth filter; cutoff frequency 0.5 Hz). For each patient, resting-state data were then epoched into consecutive segments of 2 s each (yielding 80–284 epochs per patient). For the task data set, data were epoched from 1.5 s before the onset of the two options on the screen to 5 s after patients indicated their choices by pressing a key (this corresponds to 3 s after the onset of the outcome in trials in which patients chose to gamble). Because movement artifacts were more likely during the task than during the resting-state recordings (for which patients only had to sit still), a simple artifact rejection routine was added for the task data set. Specifically, a trial was rejected if the maximum amplitude variance across all available channels in that trial exceeded a set threshold, as implemented by standard options in FieldTrip. This resulted, on average, in a rejection of 12.5% of all task-related trials.

#### Time windows for distinct task stages.

Our primary interest in the task data set was to test for a modulation of cortico-accumbens connectivity during different stages of the task. Specifically, we asked how connectivity changed as patients decided between the two options and then anticipated and subsequently saw the outcome of a chosen gamble. To compare connectivity during the decision, anticipation, and outcome stages with a baseline, we divided each trial into four consecutive time windows, each equal to 1.5 s in duration (see Fig. 2*A*). The 1.5 s preceding the onset of the two options on the screen served as a baseline (see ITI in Fig. 2*A*). The decision stage was defined as the time interval 1.5 s before patients pressed a key to choose one of the two options. Trials in which patients responded to the presentation of the options faster than 1.5 s were excluded from analysis (of all 4 task stages). Given response times in the task [medians: 2.09 s (P1), 3.04 s (P2), 1.52 s (P3), 2.11 s (P4), and 1.46 s (P6)], this time interval of 1.5 s is likely to include (aspects of) the decision process. A pre-response interval of 1.5 s also allowed us to use analysis time windows of equal duration across the four task stages (note that, across trials, the shortest ITI available as a baseline was 1.5 s). Accordingly, the anticipation stage of the task was defined as the 1.5 s that preceded the onset of the gamble outcome, whereas the 1.5 s after outcome presentation corresponded to the outcome stage.

By design, trials in which patients chose the safe option differed fundamentally from gamble trials in that, in the former, patients knew immediately the outcome of their decision. Consequently, anticipation and outcome stages do not exist in trials in which the safe option was chosen, at least not in the same sense as in gamble trials. Because the connectivity metrics used in this study are sensitive to the number of trials used for analysis, trials in which patients chose the safe option were excluded from the comparison of the four task stages (note that we report a control analysis of our main contrast of interest, comparing the decision stage with the baseline, which does include trials in which the safe option was chosen). The numbers of trials available for all four task stages, i.e., trials in which subjects chose to gamble and responded with a minimum reaction time of 1.5 s, were 66 (P1), 102 (P2), 48 (P3), 103 (P4), and 63 (P6).

#### Functional and directed connectivity.

Because the brain and surrounding tissues have no relevant capacitance across the frequency range of interest in EEG (⪡1 kHz), signals measured at surface electrodes instantaneously reflect underlying cortical generators (e.g., Penny et al. 2011; Schomer and Lopes da Silva 2012). Because of this, connectivity between LFP recorded via DBS electrodes and surface EEG can be interpreted as cortico-subcortical coupling.

We first computed the spectral coherence between the signal at Cz and the signal at each of the six accumbens channels (3 for each hemisphere). Coherence indexes the consistency of a linear phase and amplitude relation between two signals across trials (Walter 1963). Coherence is defined as the absolute value of the trial-averaged cross-spectrum, normalized by the square root of the product of the two trial-averaged autospectra. Coherence ranges from 0 to 1, with higher values indicating higher consistency of a phase and amplitude relation between two signals. Coherence was computed across trials from the cross- and autospectra after a fast Fourier transform of the Hanning-tapered data for each trial/resting-state epoch was obtained.

Because coherence intermingles phase and amplitude, we tested for significant phase synchronization between cortex (Cz) and the nucleus accumbens with a separate metric. To test for phase synchronization, the weighted phase-lag index (wPLI) was computed, a recent extension (Vinck et al. 2011) of the phase-lag index (PLI; Stam et al. 2007). The (signed) PLI is defined as the trial-averaged sign of the imaginary component of the cross-spectrum and ranges from −1 to 1. In cases where two signals show little or no (non-zero) phase synchronization, the PLI tends toward zero. Values closer to −1 or 1, on the other hand, indicate that phase lags or leads between two signals are not equiprobable across trials, thus reflecting a consistent phase relation. A further improvement in the sensitivity of the PLI has recently been demonstrated by weighting the sign of the imaginary part of the cross-spectrum by its magnitude (i.e., by the π/2 component of the phase difference between signals; Vinck et al. 2011). In the present study, the wPLI was computed on the basis of the single-trial Fourier spectra, using standard settings in FieldTrip.

To determine directionality of information flow between cortex and the nucleus accumbens, we computed a nonparametric variant of pairwise Granger causality (Dhamala et al. 2008). Pairwise Granger causality indexes the contribution of one time series to forecasting a second time series, over and above the variance already explained by the past information of the latter. In the frequency domain, the computation of Granger causality is based on the spectral transfer function and the noise covariance matrix, which can be obtained either from an autoregressive model or, alternatively, nonparametrically, from a factorization of the spectral matrix. The latter approach avoids any assumptions regarding a specific autoregressive model order and was used in the present study. Nonparametric Granger causality was computed from single-trial Fourier spectra in FieldTrip, using standard settings (i.e., a factorization based on the Wilson algorithm; Wilson 1972).

Delays between surface EEG and nucleus accumbens LFP were obtained from a regression of phase differences on frequency, following previous work (Riddle and Baker 2005; Rosenberg et al. 1989). The rationale behind a phase-frequency regression is that a constant conduction delay should lead to a linear increase in phase differences across a frequency range that shows significant coupling. Phase differences for the combination of each nucleus accumbens channel with Cz were obtained from the cross-spectra of each patient. Because we expected similar delays for all nucleus accumbens channels, phase differences were pooled across channels [this also increases the number of data points available for the regression analysis; see Riddle and Baker (2005) for a similar pooling of channels]. For each patient, phase differences were then linearly regressed on frequency across the low-frequency range that showed significant coherence in that particular patient (Fig. 1*A*). The slope of the regression line (in ms) was used as a proxy for conduction delays. Because frequency windows with significant coherence were quite narrow in some patients, particularly in P3 and P4, phase differences were computed across resting-state epochs of 6.7 s each to increase the frequency resolution, and thereby the number of frequency bins available for the regression analysis. This value was chosen to achieve a high-frequency resolution while keeping the number of epochs available for the estimation of the cross-spectrum as high as possible.

Only two patients showed a significant fit of the regression line and acceptable *R*^2^ values (both *P* < 0.0005; *R*^2^ values of 0.074 and 0.16), presumably because their resting-state recordings were longest (∼10 min each). In the other three patients (3–5 min of recordings), the fit was far from significant (all *P* > 0.2, *R*^2^ < 0.01), possibly because an insufficient number of trials were available to estimate the cross-spectrum with sufficiently low noise (≤40 trials each) and a relatively low number of frequency bins for the regression (as low as 11). Because data segments for the different task stages were only 1.5 s long, delays were only computed from the resting-state data.

**Fig. 3.**
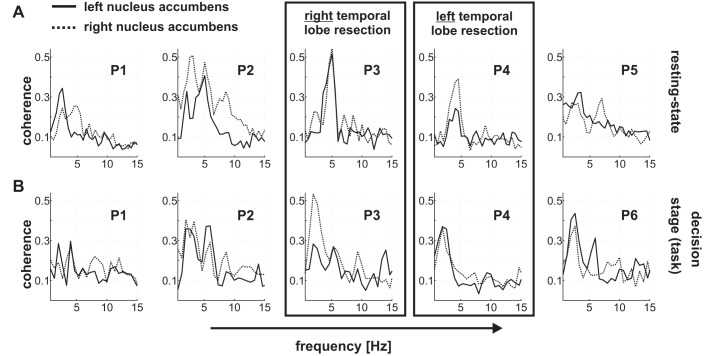
Coherence between cortex and the nucleus accumbens, separately for each hemisphere, both at rest and during the decision stage of the task. In all plots, the dotted line represents coherence spectra between cortex and the right nucleus accumbens, and the solid line corresponds to coherence with the left nucleus accumbens. *A*: coherence at rest. *B*: coherence during the decision stage of the task. The boxes mark the 2 patients (P3 and P4) who had undergone resection of the right (P3) and left (P4) medial temporal lobe in the past. Cortico-accumbens coherence is not altered systematically by unilateral resection of the hippocampus (P3 and P4).

For all connectivity analyses, the lowest frequency and the frequency resolution were defined as the inverse of the trial/epoch length in seconds. For the resting-state data set, the coherence spectra, wPLI spectra, and Granger causality spectra were explored over a broad frequency range (up to 100 Hz). Having established a frequency window of interest in our analysis of the resting-state data, we tested for a task-dependent modulation of cortico-accumbens connectivity in this frequency window (≤6 Hz). Note that this frequency window of interest was obtained from the independent analysis of the resting-state data set. Because we had no prior hypothesis regarding any difference between the left and right nucleus accumbens, or between ventral and dorsal channels, mean spectra across all six nucleus accumbens channels were used for statistical analysis. We show data for the left and right nucleus accumbens separately in Fig. 3. Separate analyses of the three DBS channels of each electrode showed no systematic differences across individuals.

### Statistics

Because of the moderate size of our study sample (5 patients for each data set), statistical group-level inference is inappropriate. Instead, we report statistical results for individual patients and note commonalities and differences between single-patient statistical results. All statistical analyses were based on nonparametric resampling tests. To test for significant peaks in the coherence and wPLI spectra, the trial order of single-trial Fourier spectra was randomly shuffled for the nucleus accumbens, but not for Cz (the same shuffled trial order was used for all 6 nucleus accumbens channels). Coherence and the wPLI were then recomputed from the shuffled data. This was repeated for at least 4,000 iterations, each with a unique, shuffled trial order for the nucleus accumbens. For each frequency bin, a *P* value was computed as the proportion of iterations whose (coherence or wPLI) values exceeded the value obtained from the original data. These *P* values were corrected for multiple comparisons across frequency bins using Bonferroni correction (between 0 and 100 Hz for the resting-state data and ≤6 Hz for the task data). Bonferroni-corrected *P* values <0.025 were considered significant (corresponding to a 2-sided test). To test for significant peaks in the coherence spectrum, a one-sided threshold (0.05) was used, because we were only interested in coherence that was larger for the original data than for the shuffled data.

To test for significant peaks in the Granger causality spectra, we compared spectra computed from the original data with Granger causality obtained after the order of time bins within each channel was reversed (effectively reversing the time axis in all channels), as recently suggested by Haufe et al. (2013). Because Granger causality is sensitive to temporal order, reversing the time axis should also reverse the direction of information flow as indexed by Granger causality. In contrast, any spurious causality, for example, due to common colored noise or differences in the signal-to-noise-ratio between channels (e.g., Nolte et al. 2008), should not be affected by a reversal of the time axis (Haufe et al. 2012). To test whether Granger causality was significantly greater when computed from the original vs. the time-reversed data, we used a nonparametric permutation test. For each trial, Fourier spectra from the original and the time-reversed data were first randomly assigned to two separate pools of trials. Granger causality was then recomputed for each pool. This was repeated for at least 1,000 iterations, each with a unique permutation. For each frequency bin, a *P* value was obtained as the proportion of permutations that resulted in a Granger causality difference that was larger than the observed difference between the original data and the time-reversed data. These *P* values were corrected for multiple comparisons across frequency bins using Bonferroni correction (between 0 and 100 Hz for the resting-state data and ≤6 Hz for the task data). Because we were only interested in Granger causality peaks that were larger for the original than for the time-reversed data, a one-sided significance threshold was used (Bonferroni-corrected *P* values <0.05).

Permutation tests were also used to test for a significant modulation of coherence during the decision-making task. We tested for changes in cortico-accumbens coherence during each of the decision, anticipation, and outcome stages of the task, each in relation to baseline. As a baseline, we used the ITI instead of our resting-state data, because the two data sets were recorded on two separate days so that potentially confounding factors, e.g., differences in tiredness, were not controlled for. Having established a frequency window of interest in the analysis of the resting-state data (≤6 Hz), *P* values were obtained from the permutation distribution after coherence values were averaged across this frequency window of interest. *P* values were Bonferroni-corrected for the number of contrasts (3) and considered significant if they fell below 0.025 (corresponding to a 2-sided test). The same procedure was used to test for differences in cortico-accumbens connectivity between the left and right nucleus accumbens and for differences between cortico-accumbens and cortico-thalamic coherence.

## RESULTS

### Cortico-Accumbens Connectivity at Rest

We first tested for significant coherence between surface-channel EEG (at Cz) and LFP from the nucleus accumbens at rest. A broad frequency range was explored (up to 100 Hz). As expected, we found significant peaks in the coherence spectrum at low frequencies (between 2.5 and 5 Hz). Remarkably, a significant low-frequency peak was present in each of the five patients (Fig. 1*A*; Bonferroni-corrected for multiple comparisons across all frequency bins up to 100 Hz). Peak frequencies and peak morphology varied across patients, with some patients (P1, P2, and P5) showing more than one distinct peak at low (delta/theta) frequencies. Up to 100 Hz, no other significant peaks were identified that were consistent across patients (P2 showed a separate peak at beta frequencies). When cortico-accumbens and cortico-thalamic coherence were compared, averaged across frequencies ≤6 Hz, cortico-accumbens coherence was significantly stronger in three patients (P2, P3, and P5; all *P* < 0.001). The remaining two patients showed trends in the same direction (P4: *P* = 0.07; P1: *P* = 0.15).

Coherence intermingles the amplitude and phase relation between two signals. To test specifically for cortico-accumbens phase synchronization, we computed the wPLI (Vinck et al. 2011), a measure of a consistent (non-zero) phase relation between two signals. Results closely resembled our coherence findings. Each of the five patients showed a significant low-frequency (3–5 Hz) peak in the wPLI spectrum (Fig. 1*B*; Bonferroni-corrected for multiple comparisons across all frequency bins up to 100 Hz). In one patient (P2), the sign of the wPLI separated three distinct peaks in the spectrum, one at delta/theta frequencies, as in all other patients, and one each at higher theta frequencies and in the beta band.

Both spectral coherence and the wPLI measure functional connectivity. To study the direction of information flow between cortex and the nucleus accumbens at rest, a nonparametric variant of spectral Granger causality was computed. Significant peaks at low frequencies (less than ∼10 Hz) were identified in the Granger causal spectrum of each patient (Fig. 1*C*; Bonferroni-corrected for multiple comparisons across all frequency bins up to 100 Hz). The direction of information flow, from cortex to the nucleus accumbens or vice versa, varied between patients. Two patients (P2 and P4) showed a predominantly subcortical drive (nucleus accumbens leading). In one patient (P1), we observed a predominantly cortical drive. One patient (P5) showed a cortical drive at ∼2.5 Hz and a subcortical drive at ∼9 Hz, and in another patient (P3), a clear distinction between directions was difficult to establish.

Delays between surface EEG and nucleus accumbens LFP were estimated from the slope of a linear regression of phase differences on frequency across the range that showed significant coherence peaks (Fig. 1*A*). Only two patients (P1 and P2) showed a significant fit of the linear regression (both *P* < 0.0005; *R*^2^ values of 0.074 and 0.16), whereas the fit was not significant in the remaining three patients (all *P* > 0.2, *R*^2^ < 0.01; see methods for a discussion of possible reasons). In P1, the estimated delay from cortex to the nucleus accumbens was +38 ms, whereas in P2, the delay was −100 ms. These delays are in line with the Granger causality results from these two patients (Fig. 1*C*). Patient P1, who showed a predominantly cortical drive, had a cortico-accumbens delay of +38 ms. The magnitude of this delay is longer than the (likely monosynaptic) conduction delays of ≤20 ms observed in rats (see discussion; McGinty and Grace 2009). In P2, on the other hand, Granger causality revealed a predominantly subcortical drive, consistent with a delay of −100 ms and a polysynaptic influence of the nucleus accumbens on cortex.

Taken together, the results of our resting-state analyses confirm that cortical EEG signals (at Cz) and LFP from the nucleus accumbens are coupled at low (delta and low theta) frequencies. At rest, the direction of this coupling varies between individuals. One possible explanation for this heterogeneity is that, by definition, resting-state recordings do not control which task a specific brain network is currently engaged in. To study how cortico-accumbens connectivity is modulated during decision making, we analyzed functional and directed connectivity during a standard decision-making task.

### Cortico-Accumbens Connectivity During Decision Making

During the task, patients chose between a certain amount of money (0 euros) and a risky gamble option that could lead to a monetary win or loss (Fig. 2*A*). Behavioral analysis confirmed that patients paid attention to the task and chose between options on the basis of the current gamble offer. Specifically, we tested whether the proportion of trials in which patients chose to gamble was higher when the mathematical expected value of the risky gamble option was relatively high vs. low (as defined by a median split). A nonparametric permutation test (1,000 unique permutations) confirmed that this was the case in four of the five patients [%gamble chosen, high vs. low expected value: 59% vs. 33% (P1), 68% vs. 53% (P2), 78% vs. 47% (P3), and 93% vs. 70% (P6); all permutation *P* values <0.025, corresponding to a 2-sided test]. Only one patient (P4) chose the gamble offer equally often when its expected value was relatively high vs. low (76% vs. 76%). Subjects earned, on average, an additional 15.66 euros (range: 3.73–27.38 euros). In contrast, a random chooser would earn an additional 8.83 euros on average, and four of the five patients earned more than that. Patients made their choices with a mean median response time of 2.09 s (range: 1.46–3.04 s). On average, they chose to gamble in 65.3% of trials. There was considerable variability in preferences across subjects (range: 46–81.5%), as expected in a task like this (Tom et al. 2007). Four of the five patients were consistent in their preference for choosing gambles, as evidenced by permutation tests that compared the tendency to choose a gamble in the first vs. second half of the experiment. Patients P1, P2, P4, and P6 chose a similar proportion of gambles in the first and second half (*P* > 0.09, threshold for significance at 0.025), whereas patient P3 chose significantly more gambles in the second than in the first half (*P* = 0.005).

We were interested how direction and strength of connectivity between cortex and the nucleus accumbens changed during the course of a trial, specifically in the low-frequency window of interest that we established in our resting-state analyses (≤6 Hz). To this end, low-frequency coherence values during the decision, anticipation, and outcome stages of the task were compared with a baseline (the ITI). Figure 2*B* shows coherence spectra during all four stages of the task (color coding corresponds to the colors in Fig. 2*A*). Four of the five patients (P2 to P6) showed at least one peak in the frequency window of interest (≤6 Hz) in at least one of the four task stages. Peak frequencies were slightly lower than at rest (around 2 Hz). Numerically, the highest coherence peak occurred during the decision stage of the task in all five patients, followed by the anticipation stage in four of the five patients. Coherence below 6 Hz was significantly higher during the decision stage compared with baseline in three of the five patients (P2, P3, and P6; all *P* < 0.025 after Bonferroni correction). The patient whose coherence and wPLI spectra at rest revealed two distinct peaks at delta and theta frequencies (P2; Fig. 1, *A* and *B*) also showed two corresponding coherence peaks during the task. When comparing the anticipation and outcome stage with baseline, we found a significant enhancement of coherence across the frequency window of interest only in one patient and one task stage (the outcome stage in P1). Trials in which patients chose the safe option (0 euros) were excluded from the analysis of task coherence to this point to avoid differences in trial numbers (see methods). However, when computing coherence across all trials and comparing the decision stage with baseline, we found a very similar pattern of results (with P2, P3, and P6 showing a significant enhancement of low-frequency coherence during the decision stage; P4 showed a trend for significance of 0.031). In contrast to this increase in cortico-accumbens coherence during the action selection stage of the task, no task modulation of cortico-thalamic coherence ≤6 Hz was observed in any of the five patients (all *P* > 0.3).

We tested whether this enhancement of functional connectivity during the decision stage coincided with a cortical drive of low-frequency oscillations in the nucleus accumbens, as predicted from the animal literature (Calhoon and O'Donnell 2013; Gruber et al. 2009a). Granger causality spectra confirmed that all patients showed a significant cortical drive at frequencies similar to their coherence peaks (except P3, who showed a trend of *P* = 0.08; Fig. 2*C*, Bonferroni-corrected for multiple comparisons across all frequency bins ≤6 Hz). For P2, there was a significant cortical drive at ∼2.5 Hz and a significant subcortical drive at ∼5.5 Hz, which, together, mapped well onto the two distinct low-frequency peaks observed in the coherence spectra of this patient at rest and during the task.

### EEG/LFP Connectivity Does Not Reflect Hippocampus/Nucleus Accumbens Coupling

Previous studies suggest that scalp recordings may reflect hippocampal signals in addition to neocortical signals (e.g., Kaplan et al. 2012). In rodents, oscillations in the hippocampus and the nucleus accumbens are coupled at theta frequencies (Berke et al. 2004; Gruber et al. 2009a). Accordingly, the EEG/LFP connectivity observed in the present study could, in principle, reflect connectivity between the nucleus accumbens and the hippocampus, rather than the neocortex. To address a potential hippocampal influence on our connectivity findings, we made use of the fact that two of the patients had undergone surgical resection of the medial temporal lobe to treat their epilepsy. Patients P3 and P4 had undergone right and left temporal lobe resection, respectively, 3 and 9 yr before DBS surgery. In nonhuman primates (unlike in rodents), hippocampal projections to the nucleus accumbens do not cross to the contralateral hemisphere (Friedman et al. 2002). Under the assumption that this holds for humans (see discussion), we tested for differences in delta/theta coherence as a function of hemisphere (operated vs. nonoperated hemisphere). If the hippocampus made a substantial contribution to the observed low-frequency coherence, we would expect reduced coherence peaks in the operated hemisphere, both at rest and during the task. If there was no substantial hippocampal contribution, removal of the hippocampus should have little impact.

Patient P4 indeed showed a significant reduction in delta/theta coherence in the left (operated) hemisphere at rest relative to the right (not operated) hemisphere (resampling *P* value <0.025). Importantly, however, this patient showed no difference between hemispheres during the decision stage of the task, where a similar coherence peak was observed in both hemispheres (Fig. 3, *A* and *B*). For patient P3, who had undergone right temporal lobe resection, we found significantly stronger coherence in the operated hemisphere during the task (resampling *P* value <0.025) and no difference at rest. In addition, patient P2, who had not undergone any surgery of the medial temporal lobe, also showed significantly stronger coherence (at rest) in the right vs. left hemisphere, similarly to P4 and P3.

## DISCUSSION

We demonstrate oscillatory coupling between LFP in the human nucleus accumbens and surface EEG at delta and low theta frequencies, both at rest and during a decision-making task. During the action selection stage of the task, this coupling consists of a cortical drive of ∼2-Hz oscillations in the nucleus accumbens, accompanied by an increase in cortico-accumbens coherence.

### Low-Frequency Oscillations and Nucleus Accumbens Network Function

Low-frequency coupling of LFP and cross-correlation of single units in the hippocampus and the nucleus accumbens are well established in rodents (Berke et al. 2004; Gruber et al. 2009a; Tabuchi et al. 2000; van der Meer and Redish 2011). Entrainment of nucleus accumbens neurons to the hippocampal theta rhythm is thought to convey contextual (spatial) information (Tabuchi et al. 2000; van der Meer and Redish 2011), possibly constraining afferent drive from prefrontal cortex during action selection (Grace 2000; Goto and Grace 2008). Conversely, recent studies have shown that prefrontal input to the nucleus accumbens can suppress excitatory drive from the hippocampus. Specifically, Calhoon and O'Donnell (2013) reported that 50-Hz train stimulation of medial prefrontal cortex attenuated excitatory postsynaptic potentials in the nucleus accumbens that were elicited via hippocampal projections. Because train stimulation is thought to mimic burst firing of prefrontal neurons during instrumental behavior (Peters et al. 2005), the authors argued that naturally occurring afferent input from prefrontal cortex, specifically during instrumental action, may also suppress hippocampal drive, possibly via inhibitory interneurons. During instrumental behavior, LFP in the nucleus accumbens core and prefrontal cortex are coupled at delta frequencies (1–4 Hz), accompanied by a decrease in theta coupling of the nucleus accumbens with the hippocampus (Gruber et al. 2009a). Taken together, these findings suggest that cortico-accumbens coupling at delta frequencies may be involved in aspects of instrumental action, at least in rodents. Translation of these findings to human physiology has so far been speculative because electrophysiological data from the human nucleus accumbens is rare.

In the present study, we demonstrated cortico-accumbens coupling at low frequencies in humans. In particular, we found a cortical drive of 2- to 3-Hz oscillations in the nucleus accumbens during the action selection stage of a standard decision-making task, accompanied by an increase in 2-Hz coherence between cortex and the nucleus accumbens. The delay between cortex and the nucleus accumbens was estimated to be ∼38 ms from a phase-frequency regression of the resting-state data of one patient (which allowed for sufficiently long epochs; see methods). This delay is longer than the (monosynaptic) conduction delays between frontal cortex and the nucleus accumbens reported in rats (≤20 ms; McGinty and Grace 2009). One possible reason for this is that cortico-accumbens coherence may involve an additional step of synaptic transmission. A plausible candidate for mediating cortico-accumbens coherence is the pool of inhibitory interneurons in the nucleus accumbens. Calhoon and O'Donnell (2013) reported evidence for an involvement of GABAergic neurotransmission in the heterosynaptic suppression of hippocampal input by prefrontal stimulation in rats, consistent with reports that prefrontal stimulation activates nucleus accumbens interneurons (Gruber et al. 2009b). Interneurons account only for a minority of striatal neurons (Jiang and North 1991), and their direct contribution to LFP (and thus to the observed cortico-accumbens coherence and estimated conduction delay) is therefore likely to be small. Taking these reports together, intermediate transmission via interneurons would agree with findings in rodents and could account for the delay between surface EEG and LFP observed in the present study. However, we note that our estimation of the conduction delay should be treated with caution because it is based on data from a single patient. Furthermore, previous studies have shown that delays computed on the basis of phase differences may not correspond well to conduction delays (Williams and Baker 2009).

A role of low-frequency oscillations in the nucleus accumbens, in the range of theta frequencies, in human decision-making has been proposed before. Cohen et al. (2009) reported a transient increase in phase synchronization at ∼4–8 Hz approximately 250–550 ms after the outcome in a reversal learning task, both between the left and right nucleus accumbens and between the nucleus accumbens and a frontal EEG electrode. In contrast to this transient phenomenon (∼1 cycle), we report cortico-accumbens connectivity over much longer segments (1.5–2 s) and at lower frequencies (2–5 Hz), which, importantly, change in strength and direction during action selection. The cortical drive consistently observed during action selection in our study is in agreement with a previous report of a Granger causal influence of cortex on the nucleus accumbens in humans (Cohen et al. 2012). However, that study focused on connectivity during outcome anticipation in a task that had no action selection component and found connectivity across a broad frequency range (of up to 20 Hz, possibly due to a limited frequency resolution, as the authors note). In contrast, we demonstrate an increase specifically in low-frequency coupling and a consistent cortical drive during the action selection stage of our decision-making task.

### Cortical Sources Involved in Low-Frequency Coupling with the Nucleus Accumbens

We studied connectivity between surface EEG recorded at electrode Cz and LFP from the nucleus accumbens. Given that surface EEG signals are subject to a high degree of volume conduction and distortion of the electric field by the skull, EEG signals at central sensors such as Cz likely contain components from widely distributed cortical sources. We addressed the possibility of a substantial contribution of hippocampal signals, recorded at surface electrodes, to the observed EEG/LFP connectivity in two patients who had undergone unilateral resection of the medial temporal lobe in the past. Using anterograde and retrograde tracer techniques, Friedman et al. (2002) demonstrated that anatomical connections between the hippocampus and the nucleus accumbens in macaque monkeys do not cross to the contralateral hemisphere. We are not aware of any anatomical studies in humans that have addressed this issue. Assuming that these results also hold in humans, we tested whether ipsilateral removal of the hippocampus systematically reduced the observed EEG/LFP coherence. Because this was not the case, a substantial hippocampal contribution seems unlikely. It is possible, however, that the nucleus accumbens of the operated hemisphere is indirectly coupled with the contralateral hippocampus, e.g., via coupling between the nucleus accumbens of each hemisphere (Cohen et al. 2009). Note that a neocortical contribution to the observed EEG/LFP connectivity does not imply that a single cortical source is consistently involved. Indeed, we find differences in coupling between our resting-state data and the task data, both in peak frequencies (which are slightly lower during the task) and in direction (with a consistent cortical drive during the task, but not at rest). This may indicate that slightly different cortical sources or neuronal populations are involved in cortico-accumbens coupling as a function of the task. Future studies that combine high-density EEG or MEG with simultaneous DBS recordings may reveal the precise cortical topography of this coupling.

### Limitations

Invasive recordings from the human nucleus accumbens are only available when there is a clinical indication for DBS therapy. In this work, we studied data recorded from patients with epilepsy. All patients underwent video EEG monitoring, which documented a focal (i.e., cortical) epileptogenic zone and excluded other epilepsy syndromes. In animal models (Deransart et al. 2000), as well as in epilepsy patients (Schmitt et al. 2014), the nucleus accumbens can be involved in seizure propagation. Accordingly, the clinical rationale underlying DBS of the nucleus accumbens in epilepsy is a suppression of seizure propagation, not of an epileptogenic focus. However, we cannot exclude the possibility that the nucleus accumbens functions differently in these patients compared with healthy individuals. During recordings, patients were constantly under supervision. No seizure was observed or reported in any case. In addition, no interictal epileptic activity was observed in the recorded data. Epileptic activity in the nucleus accumbens as a potential confounding factor can therefore be excluded.

It is important to note that all six patients were on treatment with anticonvulsant drugs during data acquisition (Table 1). The predominant mechanisms of action of these drugs consist either in a blockade of ion channels (lacosamide, lamotrigine, zonisamide, carbamazepine, oxcarbazepine) and/or in an interference with GABAergic neurotransmission (stiripentol, clozabam). Although we cannot rule out the possibility that these drugs changed cortico-accumbens connectivity, we note that the heterogeneity in medication across patients (Table 1) contrasts with the high consistency of our findings. In particular, patients whose medication involved drugs known to enhance or mimic GABAergic neurotransmission (especially clobazam and stiripentol in P2, possibly also carbamazepine in P2 and P3) showed similar results to all other patients, whose medication acts primarily via ion channels [lacosamide and lamotrigine in patients P4, P5, and P6; but see Cunningham and Jones (2000) for a potential GABAergic enhancement by lamotrigine, although this has been questioned, see Braga et al. (2002)].

**Table 1.** Patient data

Patient	Sex/Age/Disease Duration, (M/F)/yr/yr	Epilepsy Syndrome	Etiology	Seizure Lateralization	Seizure Onset	Antiepileptic Drug	Surface EEG Channel, resting state/task
P1	M/39/9	Multifocal	Cryptogenic	Bilateral	Bifrontal	LCM (400 mg), ZNS (400 mg)	FCz, Cz/Oz, Fpz, Cz
P2	M/32/31	Multifocal	Genetic (SCNA1)	Left, possibly bilateral	Bifrontal and left mediotemporal	OXC (900 mg), clobazam 5 mg), STP (4,500 mg)	T1, T2, Fpz, Oz, Cz/Oz, Fpz, Cz, POz
P3	F/44/14	Multifocal	Right hippocampal sclerosis*	Bilateral	Temporal	CBZ (1,200 mg)	Oz, Fpz, Cz/Oz, Fpz, Cz
P4	M/40/31	Focal	Left hippocampal sclerosis†	Left	Temporal	LTG (400 mg), LCM (400 mg)	Oz, Fpz, Cz/Oz, Fpz, Cz
P5	F/28/12	Multifocal	Cryptogenic	Bilateral	Temporal	LTG (200 mg, LCM (200 mg)	Fpz, Cz/no task data
P6	F/52/17	Focal	Cryptogenic	Left	Temporal	LTG (250 mg), LCM (400 mg)	No resting-state data/Oz, Fpz, Cz

M/F, male/female; LCM, lacosamide; ZNS, zonisamide; OXC, oxcarbazepine; STP, stiripentol; CBZ, carbamazepine; LTG, lamotrigine.

* P3 underwent right temporal lobe resection 3 yr before deep brain stimulation (DBS) surgery.

† P4 underwent left temporal lobe resection 9 yr before DBS surgery.

Our study depends on a precise placement of DBS electrodes into the nucleus accumbens. Despite high surgical expertise, we cannot fully exclude slight imprecision in electrode placement. Because safety considerations forbid postsurgical MRI, the location of the electrodes can only be assessed by combining presurgical MRI with postsurgical CT. Following this approach, coordinates of the most ventral contacts in our study, averaged across all patients, are [7 6 −9] (right hemisphere) and [−7 6 −9] (left hemisphere; normalized to MNI space). However, this analysis is subject to some inaccuracy itself, because it relies on two separate imaging techniques and data from two different days.

We cannot exclude the possibility that discrete structural changes to the nucleus accumbens during electrode implantation, such as trauma or edema, may have influenced coherence and Granger causality spectra. Structural changes due to electrode implantation are thought to be responsible for the so-called “stun effect,” i.e., a transient amelioration of symptoms in some patients with Parkinson's disease in the first days after DBS surgery, before the stimulator is switched on (e.g., Eusebio and Brown 2009). However, it is difficult to explain a frequency-specific task modulation, as demonstrated in our study (Fig. 2), purely on the basis of such structural alterations.

In summary, we demonstrate highly consistent oscillatory coupling between cortex and the human nucleus accumbens at delta and low theta frequencies. During action selection, this coupling turns into a cortical drive of delta oscillations in the nucleus accumbens. In view of a similar coupling in rodents during instrumental behavior, and in light of the high consistency of our findings across subjects, cortico-accumbens delta coupling may represent a highly conserved mechanism of regulating neuronal excitability and, ultimately, action selection in the nucleus accumbens.

## GRANTS

This work was supported by Wellcome Trust Ray Dolan Senior Investigator Award 098362/Z/12/Z. M.-P. Stenner was supported by Deutsche Forschungsgemeinschaft scholarship STE 2091/1-2. R. B. Rutledge was supported by the Max Planck Society. T. Zaehle, J. Voges, and H.-J. Heinze received funding from Deutsche Forschungsgemeinschaft Sonderforschungsbereich Grant SFB-779 TPA2. The Wellcome Trust Centre for Neuroimaging is supported by core funding from the Wellcome Trust 091593/Z/10/Z.

## DISCLOSURES

No conflicts of interest, financial or otherwise, are declared by the authors.

## AUTHOR CONTRIBUTIONS

M.-P.S., V.L., and R.B.R. conception and design of research; M.-P.S., V.L., and R.B.R. analyzed data; M.-P.S. and V.L. interpreted results of experiments; M.-P.S. prepared figures; M.-P.S. drafted manuscript; M.-P.S., V.L., R.B.R., F.C.S., J.V., and R.J.D. edited and revised manuscript; M.-P.S., V.L., R.B.R., T.Z., F.C.S., J.V., H.-J.H., and R.J.D. approved final version of manuscript; T.Z., F.C.S., and J.V. performed experiments.
